# Knowledge and practices about vocal hygiene among speech-language pathologists in India

**DOI:** 10.1371/journal.pone.0334501

**Published:** 2025-10-15

**Authors:** Anubhuti Jain, Divya Seth, Dhanshree R. Gunjawate

**Affiliations:** 1 Department of Audiology and Speech Language Pathology, Kasturba Medical College Mangalore, Manipal Academy of Higher Education, Manipal, India; 2 Department of Speech Language Pathology, All India Institute of Speech and Hearing, Mysore, Karnataka, India; Father Muller Charitable Institutions, INDIA

## Abstract

**Background:**

The objective of the present study was to explore the knowledge and practices about vocal hygiene among speech language pathologists in India.

**Methods:**

An online survey was conducted to explore the knowledge and practices about vocal hygiene among speech language pathologists in India. A questionnaire was developed based on expert opinion and literature and included questions on demographic details, vocal hygiene knowledge and practices. Descriptive statistics was used to summarize continuous variables, and discrete variables were analysed using frequency and percentages.

**Results:**

A total of 123 responses were considered for analyses. 74.8% of the speech language pathologists self-reported of experiencing more than one vocal symptom/difficulty. The speech language pathologists displayed good knowledge of the vocal and non-vocal factors affecting voice. With respect to reported practice, speech language pathologists exhibited inconsistencies in practice. Despite being aware about the vocal hygiene practices, speech language pathologists did not consistently implement them in daily routines.

**Conclusion:**

The speech language pathologists are knowledgeable about different factors that have a positive and negative influence on voice; however, they do not practice everything regularly. As SLPs play a dual role of being a professional voice user and a clinician, it is important that they practice what they teach their patient.

## Background

Globally, over one third of the entire workforce depends on their voice to fulfil their professional demands [1–3]. Professional voice users (PVUs) are completely or partially dependent on their voice to fulfil their professional requirements and a consistent quality of voice. These include actors, singers, teachers, clergy, attorneys, physicians, politicians, receptionists, telemarketers, telephone operators among others [4,5]. The vocal requirements of PVUs vary across professions, with differences in vocal load, loudness and voice quality needed to suit their professional roles [6]. PVUs have unique vocal requirements based on the demands of their profession and any alteration in their voice can impact their job and thereby productivity at work. They are often required to use their voice at higher loudness levels and variable pitches for prolonged durations [7], placing them at a higher risk of developing voice problems. Voice problems can have a negative impact on their work, efficiency, quality of life, participation leading to financial losses and absenteeism. This can impact their overall sense of well-being and their sense of self [8,9].

PVUs can achieve and sustain good vocal health by professional guidance and implementing vocal hygiene practices [5,10,11]. A vocal hygiene program refers to a comprehensive framework encompassing various aspects of vocal healthcare. It is an indirect patient-centred intervention and includes adoption of strategies to help maintain a healthy and powerful voice [10]. A comprehensive vocal hygiene program often recommended by a speech-language pathologist (SLP), encompasses education on the physiology of the phonatory system, identification of phono traumatic behaviours, reduction of these behaviours, implementation of voice rest or modified voice rest, management of high-risk vocal situations, regulation of optimal loudness and pitch levels, adequate hydration, and modifications in lifestyle [10–12]. SLPs play an integral role in the implementation of these programs, who educate and guide individuals to integrate the vocal hygiene strategies that promote vocal well-being.

In India, the field of Audiology and Speech Language Pathology formerly established in 1960s is under the regulation of the Rehabilitation Council of India (RCI). The RCI serves as a regulatory body for diploma, undergraduate and postgraduate courses. SLPs in India often manage large caseloads from varied cultural, linguistic, ethnic, and socioeconomic backgrounds. SLPs in India often work in a variety of settings such as clinics, hospitals, nursing homes, medical centres, schools, multidisciplinary centres, university/academic institutions, hearing aid and cochlear implant companies, private clinics and tele practice clinics with most working in more than one work setting and performing multiple roles. Challenges such as inadequate manpower, occupational stress, diversity in clinical population, insufficient monetary compensation, poor awareness about the profession, lack of updated professional training opportunities lead to poor work satisfaction [13–15]. A recent survey in audiologists and SLPs in India exploring the workforce demographics and patterns in practice reported that 72.8% respondents worked over 7 hours, 44.1% reporting moderate vocal efforts while 29.8% experiencing severe strain on their busiest workdays [16]. Challenges related to workloads, manpower, adverse work conditions such as dryness, humidity, dust or noise often led to high vocal demands.

Further, the SLPs rely heavily on their voice for professional commitments such as to assessment, counselling patients, giving therapy, explaining and demonstrating exercises and techniques. Studies in SLPs across the world have revealed that although they are knowledgeable and strong advocates of good vocal hygiene, they struggle to consistently apply these for themselves [17–21]. Studies in SLPs across the world have revealed the high levels of vocal load [16,19,22–24] and thereby vocal fatigue experienced by the SLPs and inconsistencies in practices to protect vocal heath [17–20,25].

In India, the SLPs face additional challenges of demanding work conditions due to high case load, patient diversity, professional challenges, and work and environmental conditions, and work across multiple settings. These factors further exacerbate the vocal load that further impacts their vocal health. Considering their responsibility as a clinician, advocate for vocal hygiene and safeguarding their own vocal health, examining their knowledge and exploring their practices is crucial. The present study aims to fill the existing gap by examining the knowledge and practices of vocal hygiene among SLPs in India. Identifying. the gaps in knowledge and practices, can help in improving training, professional standards and advocacy for better vocal hygiene and vocal health within the profession.

## Methods

A descriptive cross-sectional questionnaire-based study was carried out to explore the knowledge and practices about vocal hygiene among SLPs practising in India. The study was approved by the ethics committee of the Kasturba Medical College, Mangalore by approval number: IEC KMC MLR 05–2022/182. Informed consent was carefully obtained from each participant before their involvement in the study. The study was conducted between 1^st^ June 2022–30^th^ June 2023.

### Questionnaire development and validation

Based on the previous literature [21,23–25] and expert opinion, a self-reported questionnaire was developed pertaining to the knowledge and practice of vocal hygiene among SLPs for the purpose of this study. The developed questionnaire was content validated by three practising SLPs with over five years of work experience in voice science and disorders All experts had doctoral degrees, conducted research in voice and were well-versed with questionnaire development methodology.

Each question was graded on a four-point scale: not relevant, somewhat relevant, quite relevant, and relevant. The items rated as relevant and quite relevant were retained in the final questionnaire. Scale-level Content Validity Index − Universal Agreement (S-CVI/UA) score was calculated to establish content validity. A score of 0.90 was obtained which indicated an excellent content validity [26] for the developed questionnaire. The questionnaire was developed and administered in the English language. No items were deleted during the content validation. The final questionnaire comprised of questions to elicit demographic details (11 items), knowledge of vocal hygiene (21 items), and vocal hygiene practices (22 items). The questionnaire is available upon request from the corresponding author.

### Participants

The participants of the study included professional SLPs practising across different work settings in India. Speech-language pathologists with a minimum of a bachelor’s degree in audiology and speech-language pathology, with minimum of one year of work experience and registered under the Rehabilitation Council of India were included. Students currently enrolled in graduate and post graduate courses of audiology and speech-language pathology were excluded from the study. Speech-language pathologists currently practising outside India were excluded.

### Procedure

The developed questionnaire was converted into a Google Form. To maintain anonymity, no personal identifiers such as name, contact data or email id were collected. A list of registered speech-language pathologists was sought from the official website of Rehabilitation Council of India. An email request was sent to all registered speech-language pathologists to participate in the study along with the details of the current research. An overview of the study’s objectives and the selection criteria were provided at the start of the Google Form. Only those speech-language pathologists who gave their written consent could participate in the study and had to click on ‘submit’ in the final section to submit their responses. SLPs who did not provide their consent to participate were redirected to the final section of the Google Form, allowing them to exit the survey without participating. All responses were saved to Google Drive and were accessible only to the investigators of the study. The participating SLPs were encouraged to forward the link to other fellow SLPs who suited the inclusion criteria. The questionnaire took about 8–10 minutes for each participant to complete*.*

### Statistical analysis

The S-CVI was calculated to establish content validity for the items in the questionnaire. Descriptive statistics was performed to summarise the demographic variables. Continuous variables were analysed using mean, standard deviation, and range while the discrete variables were analysed using frequency and percentages. The percentage of SLPs who correctly and incorrectly identified the influence of each of the factors on vocal hygiene was computed. Responses to the open-ended question on vocal and non-vocal practices for vocal hygiene were listed and the frequency of responses were calculated. All statistical analysis was done using IBM SPSS Statistics Version 25 [27].

## Results

A total of 142 responses were received out of which 19 were excluded owing to incomplete responses or if the respondent had less than a year of work experience. A total of 123 responses were considered for further analyses. The demographic details of the SLPs have been depicted in Table 1.

**Table 1:** Demographic details.

		Mean ± SD	Range
**Age (in years)**		31.10 ± 6.29	22 - 60
**Years of experience (in years)**		7.08 ± 5.87	1 - 36
		**Number (*n*)**	**Percentage (%)**
**Work experience (in years)**	<1–2 years	24	19.5
	>2–5 years	33	26.8
	>5–10 years	40	32.5
	>10 years	26	21.1
**Gender**	Male	30	24.4
	Female	91	74
	Prefer not to say	2	1.6
**Degree**	BASLP	50	40.7
	MASLP/MSLP	67	54.5
	PhD	6	4.9
**Work setting**	Academic	24	19.5
	Clinical	59	48
	Hospital	40	32.5
**Age group to whom services is provided**	Paediatric	71	57.7
	Adult	45	36.6
	Geriatric	7	5.7
**Caseload**	Childhood language disorders	22	17.9
	Adult Language Disorders	4	3.3
	Hearing Loss	1	0.8
	Fluency	2	1.6
	Voice	6	4.9
	Motor Speech Disorders	2	1.6
	More than one	84	68.29

As shown in Table 1, the average age of the participants was 31.10 ± 6.29 years, with an average work experience of 7.08 ± 5.87 years. Most of the participants (76.4%) reported that they worked five to seven days per week, while a few others (23.6%) worked three to five days per week. In. terms of work hours, 56.1% reported working six to eight hours per day 26% worked eight to ten hours per day while a relatively smaller proportion (17.9%) worked only four to six hours per day. The session duration varied, with 25.2% reporting conducting a 30-minute session, 70.7% reporting 45-minute session while only 4.1% conducting a 60-minute session.

Among the vocal symptoms/difficulties reported by SLPs, 74.8% experienced multiple difficulties. Among the symptoms, 13.8% reported dryness, followed by voice break (4.1%) and tightness in throat, neck, or shoulders (2.4%). Loss of loudness, loss of pitch range, pain and hoarseness were isolated symptoms experienced by only 0.8% respondents, while none reported harshness or breathiness as an isolated symptom.

### Knowledge of vocal hygiene

The questionnaire included 21 items related to knowledge of vocal hygiene among SLPs which they had to identify as having positive, negative or no influence on voice. Frequency analysis was done to determine the percentage of SLPs who indicated positive, negative or no influence for each of the factors as displayed in Table 2. The option highlighted in bold is the correct/expected response.

**Table 2:** Factors affecting vocal hygiene*.*

	Positive influence *n* (%)	No influence *n* (%)	Negative influence *n* (%)
Being happy	**108 (87.8)**	14 (11.4)	1 (0.8)
Being relaxed	**117 (95.1)**	6 (4.9)	–
Drinking 8–10 glasses of water	**120 (97.6)**	2 (1.6)	1 (0.8)
Good posture	**117 (95.1)**	5 (4.1)	1 (0.8)
Resting my tired voice	**111 (90.2)**	5 (4.1)	7 (5.7)
Speaking gently	**110 (89.4)**	9 (7.3)	4 (3.3)
Steam inhalation	**101 (82.1)**	19 (15.4)	3 (2.4)
Using an amplifier or microphone	**90 (73.2)**	20 (16.3)	13 (10.6)
Warming up my voice before talking	**105 (85.4)**	14 (11.4)	4 (3.3)
Being overweight	3 (2.4)	38 (30.9)	**82 (66.7)**
Consumption of spicy and oily food	11 (8.9)	12 (9.8)	**100 (81.3)**
Drinking alcohol	2 (1.6)	25 (20.3)	**96 (78)**
Drinking coffee, tea, or soda	16 (13)	27 (22)	**80 (65)**
Noisy environment	3 (2.4)	10 (8.1)	**110 (89.4)**
Shouting	4 (3.3)	6 (4.9)	**113 (91.9)**
Smoking	2 (1.6)	14 (11.4)	**107 (87)**
Throat clearing	10 (8.1)	10 (8.1)	**103 (83.7)**
Whispering	8 (6.5)	16 (13)	**99 (80.5)**
Eating warm food	61 (49.6)	**45 (36.6)**	17 (13.8)
Swimming	31 (25.2)	**78 (63.4)**	14 (11.4)

*response in bold is the correct and expected response.

As noted from Table 2, most participants correctly identified the factors with positive and negative influences on voice. However, the responses were varied for the two factors which did not influence voice such as eating warm food and swimming. More than 80% of the SLPs correctly identified factors with positive influence such as being relaxed, drinking 8–10 glasses of water, good posture, resting tired voice, warming up voice before talking, steam inhalation, and speaking gently. Among the factors with a negative influence, shouting, consumption of spicy and oily food, noisy environment, smoking, throat clearing and whispering were correctly identified by more than 80% of the SLPs.

### Vocal hygiene practices among SLPs

The SLPs were provided with 20 common vocal hygiene practices consisting of non-vocal and vocal behaviours known to have an influence on voice and were asked to indicate how often they followed them using a four-point scale – always (100% of time), sometimes (50% of time), rarely (25% of time), and never (0% of time). Their responses have been tabulated in Table 3.

**Table 3:** Vocal Hygiene Practices reported by the SLPs*.*

	Always – (100% of time) *n* (%)	Sometimes (50% of time) *n* (%)	Rarely (25% of time) *n* (%)	Never (0% of time) *n* (%)
***Non-vocal behaviours***				
7-8 hours of sleep	50 (40.7)	61 (49.6)	12 (9.8)	–
8-10 glasses of water	61 (49.6)	51 (41.5)	11 (8.9)	–
Avoiding oily food	10 (8.1)	85 (69.1)	25 (20.3)	3 (2.4)
Avoiding spicy food	23 (18.7)	64 (52)	32 (26)	4 (3.3)
Chewing tobacco	5 (4.1)	1 (0.8)	29 (23.6)	88 (71.5)
Drinking alcohol	6 (4.9)	9 (7.3)	34 (27.6)	74 (60.2)
Consuming more than 3 cups of tea/coffee per day	17 (13.8)	28 (22.8)	44 (35.8)	34 (27.6)
Sleeping immediately after dinner	8 (6.5)	45 (36.6)	35 (28.5)	35 (28.5)
Smoking	4 (3.3)	4 (3.3)	30 (24.4)	84 (68.3)
Steam inhalation	11 (8.9)	49 (39.8)	48 (39)	15 (12.2)
***Vocal behaviours***				
Avoiding voice usage for prolonged durations	18 (14.6)	74 (60.2)	29 (23.6)	2 (1.6)
Gargling	8 (6.5)	43 (35)	53 (43.1)	19 (15.4)
Practicing abdominal breathing	25 (20.3)	35 (28.5)	43 (35)	20 (16.3)
Speaking for long hours	22 (17.9)	60 (48.8)	25 (20.3)	16 (13)
Taking break between therapy sessions	24 (19.5)	70 (56.9)	24 (19.5)	5 (4.1)
Talking in low pitched voice for long durations	3 (2.4)	40 (32.5)	46 (37.4)	34 (27.6)
Talking while having laryngitis or throat infection	3 (2.4)	35 (28.5)	55 (44.7)	30 (24.4)
Treating acid reflux/ heartburn	17 (13.8)	34 (27.6)	38 (30.9)	34 (27.6)
Performing vocal cool down exercises after session	11 (8.9)	36 (29.3)	43 (35)	33 (26.8)
Performing vocal warm up exercises before starting session	22 (17.9)	35 (28.5)	37 (30.1)	29 (23.6)

As seen in Table 3, the non-vocal behaviour most practiced always by nearly half of the respondents was consuming 8–10 glasses of water. Further, several non-vocal behaviours were practiced sometimes by a substantial proportion of SLPs. These included 7–8 hours of sleep (49.6%), avoiding oily (69.1%) and spicy (52%) foods. Most SLP reported never engaging in harmful non -vocal behaviours such as chewing tobacco (71.5%), drinking alcohol (60.2%) and smoking (68.3%). Scattered responses were noted for non-vocal behaviours that influence voice such as steam inhalation, consuming more than 3 cups of tea/coffee per day and sleeping immediately after dinner.

Overall, none of the vocal behaviours were consistently practiced always by more than 25% respondents indicating that the SLPs reported of practicing them only sometimes or rarely. Avoiding voice usage for prolonged durations (60.2%), taking break between therapy sessions, (56.9%), was most practiced sometimes. Several vocal behaviours that are beneficial to the voice, were reported of being rarely practiced by a substantial proportion of SLPs. These included talking while experiencing laryngitis or throat infection (44.7%), gargling (43.1%), talking in a low-pitched voice for extended periods (37.4%), practicing abdominal breathing (35%), managing acid reflux or heartburn (30.9%), performing vocal warm-up (30%) and cool-down exercises (35%).

In addition to the vocal hygiene practices included in the questionnaire, participants were asked an open-ended question to share any other vocal hygiene practices they follow. The responses obtained are summarised in Table 4 below.

**Table 4:** Additional Vocal Hygiene Practices reported by the SLPs.

Additional Vocal Hygiene Practices	Number (*n*)	Percentage (%)
Voice rest	6	4.8
Breathing exercise	4	3.2
Avoiding chilled or cold items	3	2.4
Relaxation exercise	3	2.4
Humming	2	1.6
Singing	2	1.6
Avoiding inhaling chemicals	1	0.8
Confidential voicing when talking for longer durations	1	0.8
Consumption of drinks with ginger	1	0.8
General fitness to facilitate flexibility of body	1	0.8
Neck and shoulder range of movements	1	0.8
Practicing trills	1	0.8
Resonant Voice Therapy	1	0.8
Vocal Function Exercises	1	0.8
Vocal laryngeal massage	1	0.8
Yoga	1	0.8

**Fig 1:**
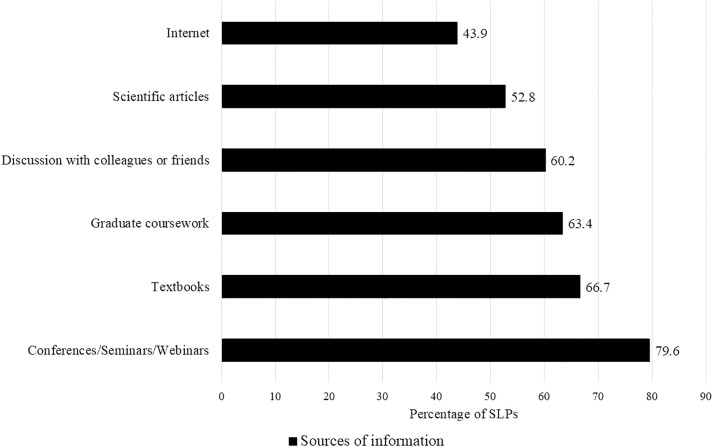
Sources used by the SLPs for information on vocal hygiene.

In the final question, the SLPs were asked to indicate the sources that they referred to gain or enhance their knowledge regarding vocal hygiene practices. The majority of the SLPs (92.7%) attributed their knowledge about vocal hygiene and practices to multiple sources such as conferences/seminars/webinars, graduate coursework, scientific articles, textbooks, internet sources, and discussions with friends or colleagues. Fig 1 presents the percentage of SLPs referring to each source.

## Discussion

Maintaining good vocal hygiene is integral to vocal health and is of particular importance to PVUs, who rely heavily on their voice to meet their occupational demands [2,4,9]. Studies consistently highlight the high predisposition of PVUs to develop voice problems due to poor vocal hygiene and high vocal demands leading to poor work efficiency and thereby impacting their quality of life [7–10]. SLPs as both advocates and healthcare providers for good vocal health, also qualify as PVUs given the heavy demands they place on their voice during assessment, counselling, teaching and therapy sessions [10,17]. Thus, the onus of maintaining a balance of taking care of their own vocal well-being while guiding patients towards healthy vocal practices. Within this context, it is important to examine the knowledge and practices of vocal hygiene among SLPs in India give the unique work conditions in which they work.

The demographic profile of the SLPs who participated in this study, with a mean age of 31.1 years which is consistent with reports on a relatively younger work force in India [13,16] with about 74% being females, which is reflective of the global trend of female predominance in the profession [28]. Most respondents (76.4%) reported working six to eight hours per day across multiple settings, handling cases across age range and variety of disorders, which highlights the diversity of the clinical responsibilities of the SLPs working in India. A high proportion of SLPs (74.8%) reported of experiencing multiple voice symptoms such as dryness, effortful speech, voice break, reduced loudness, pain in the throat, hoarseness, harshness, breathiness, reduced pitch range, and tightness in throat, neck, or shoulders. These align with previous findings among PVUs including SLPs being at a high risk of developing voice problems due to workload, working conditions, environmental factors and limited recovery time [4,9,10,17,18].

Most respondents demonstrated an adequate knowledge of factors having a positive or negative influence on voice. Over 80% SLPs correctly identified positive influences such as relaxation, adequate hydration, posture, voice rest, vocal warm-up, steam inhalation and speaking gently. A similar proportion also correctly identified the negative influences such as shouting, speaking in noisy environment, consumption of oily or spicy food, clearing the throat, whispering, smoking. These align with well-established evidence-based recommendations for vocal hygiene [4,7,9,10], highlighting awareness among the SLPs about what should be adopted or avoided for optimal vocal health. The variabilities in the voice care practices, lack of a structured vocal hygiene program, influence of cultural beliefs, anecdotal or personal experiences brings about a variability in the components of a vocal hygiene program [2,10,11,29]. To overcome these variabilities and challenges, it is thus crucial to have a more standardized and evidence based vocal hygiene program that can be adapted based on clinical, research or educational contexts.

Interestingly, while the knowledge of vocal hygiene among SLPs appeared to be adequate as noted from their ability to correctly identify the positive and negative factors affecting voice, the transition of these into practice was less evident. These discrepancies between having the knowledge and practicing it has also been reported in prior research [17,18,23,29].

Non-vocal behaviours such as ensuring 7–8 hours of sleep and 8–10 glasses of water were practiced always by more than 40% of the SLPs. These practices are based on well-established recommendations included in vocal hygiene that emphasize the importance of adequate hydration [30] and rest [31]. This high-quality evidence in support of vocal hydration emphasizes on importance of systemic hydration as the most cost-effective and easiest solution for improving voice quality [30]. A majority of SLPs reported that they never indulge in tobacco chewing, alcohol intake or smoking, all of which are well-established risk factors that can negatively impact vocal health [32–34]. It is encouraging that SLPs are aware about the detrimental and harmful effects of these agents on voice and thus refrain from their use. However, practices like limiting caffeine intake, avoiding oily and spicy food and steam inhalation received diverse response. These could be reflective of the variability based on evidence, cultural beliefs, or personal experiences in the absence of stronger evidence-based guidelines.

Under the vocal behaviours, no single practice was reported to be consistently practiced always by more than 20% of the respondents. This could be indicative of the gaps in adoption of these practices or practical difficulties in following them in day-to-day life. Avoiding speaking for long duration and taking breaks between sessions were reportedly practiced sometimes (i.e., 50% of the times) by the SLPs. Vocal hygiene habits such as gargling, vocal warm-up and cool-down exercises were rarely practiced by a majority. Gargling has been investigated to be beneficial in treatment of muscle tension dysphonia while warm-ups and cool-downs help improve voice quality and performance [35–37]. Warm-ups and cool-downs are commonly suggested by SLPs to PVU, however, prior studied have also indicated a less preference among them to practice themselves [17,38]. Positive practices such as abdominal breathing, treating reflux and negative practices such as speaking long hours, talking while having laryngitis, received diverse responses. The common factors such as speaking long hours and talking while having laryngitis are indicative of the professional retirements of SLPs that force them to continue using their voice. The variability in the responses towards practices once again highlight the discrepancies in the practical aspects of the vocal hygiene. The responses to the open -ended question on use of any other vocal hygiene practices yielded a wide range of responses. These included voice rest, breathing exercises, avoiding cold/chilled items, relaxation exercise and humming to name a few. All the responses were known factors having a positive influence on the voice.

## Future directions

The findings highlight the urgent need of having better evidence based structured vocal hygiene programs for better uptake and consistency. Efforts need to be concentrated on developing modules for academic and clinical training, focus on the curriculum and programs directed at continuing education programs. The diversity faced by SLPs in India is unique and these need to be explored in the context and applicability of such programs. Longitudinal studies can help evaluate the effectiveness of these programs on the vocal health, professional performance and their quality of life. Advocacy at the levels of clinical settings, institutions and other workplaces is needed to audit the existing vocal load by ensuring manageable caseloads, scheduled breaks, bureaucratic support and access to resources and help whenever required.

## Limitations

The use of a self-reported questionnaire might have introduced some response and recall bias. The cross-sectional nature of this enquiry provides an overview of the responses when they were collected and prevents from establishing causality or long-term effects. The use of survey limited the responses and the use of in-depth interviews supported with observation would have provided more in-depth information. The study was limited to vocal needs of SLPs professional commitments and did not extend to use of voice for other activities.

## Conclusion

The findings of the present study highlight SLPs being knowledgeable about the positive and negative influences on voice. However, they exhibited inconsistencies in putting it into practice. Factors such as time constraints due to commitments at work, workload, access to resources and overall lack of structured vocal hygiene program that is evidenced based may contribute to these gaps. As SLPs play a dual role of being a PVU and a clinician, it is important that they practice what they teach their patient. This will help in safeguarding the vocal health and the overall wellbeing of the SLPs and enable them to effectively promote vocal well-being for all.
